# Questionnaire survey on the usage of antiseizure drugs for dogs and cats in Japanese veterinary hospitals (2020)

**DOI:** 10.1002/vms3.810

**Published:** 2022-04-20

**Authors:** Satoshi Mizuno, Rikako Asada, Daisuke Hasegawa

**Affiliations:** ^1^ Laboratory of Veterinary Radiology Nippon Veterinary and Life Science University Tokyo Japan; ^2^ The Research Center for Animal Life Science Nippon Veterinary and Life Science University Tokyo Japan

## Abstract

Epilepsy is the most common neurological disorder in veterinary medicine. Recently, evidence‐based recommendations or systematic reviews for using antiseizure drugs (ASDs) in dogs and cats have been published, but there are many differences in economic, geographical, and historical backgrounds and/or the availability of each ASD between countries. In the present study, we conducted a questionnaire survey on the usage of ASDs in 511 veterinary hospitals in Japan in 2020. As a result, zonisamide (ZNS) was the most commonly prescribed drug for idiopathic (83%) and structural epilepsy (76%) in dogs. In cats, phenobarbital was the most frequently prescribed drug for idiopathic (48%) and structural epilepsy (51%), but ZNS was also commonly prescribed (41% and 36%, respectively). Additionally, ZNS was the most frequently used ASD in combination therapy for canine idiopathic epilepsy. We also surveyed the frequency of measuring ASD blood levels; however, a relatively high percentage of hospitals (22%) did not perform such measurements. Although the evidence level for ZNS is still poor for both species, it is generally used as the first‐line ASD in Japan. A large‐scale and higher evidential study for ZNS and the education of practitioners for adequate antiseizure medication are required.

Epilepsy is the most common neurological disorder in dogs and cats, which is characterized by recurrent epileptic seizures caused by various aetiologies. With reasonable seizure control, especially in idiopathic epilepsy, a relatively good and long‐term prognosis can be expected with adequate antiseizure drug (ASD) therapy, which is known as antiseizure medication (ASM) (Hamamoto et al., 2016). In veterinary medicine, phenobarbital (PB) and potassium bromide (KBr) have been popular due to their long history, widespread availability and low cost. Besides, since the 1990s, as the second generation of ASDs, such as levetiracetam (LEV), zonisamide (ZNS), felbamate, topiramate, gabapentin (GBP) and pregabalin (PGB), has been developed and approved in human medicine, veterinarians have begun to use these drugs for their patients. Most recently, imepitoin (IMP) has been approved as a new generation ASD in veterinary medicine. For dogs with epilepsy, the International Veterinary Epilepsy Task Force (IVETF) (Bhatti et al., 2015) and the American College of Veterinary Internal Medicine (ACVIM) (Podell et al., 2016) proposed consensus statements and recommendations for ASD use based on previously published studies including systematic reviews (Charalambous et al., 2016, 2014). These consensuses recommend that ASM should be initiated in dogs with (1) ≥2 seizures within a 6‐month period; (2) cluster seizures (or acute repetitive seizures) or status epilepticus; (3) the presence of structural lesions; and (4) prolonged, severe or unusual postictal period. Conversely, a consensus report for ASD usage in cats with epilepsy has not been published, but several reviews and research papers for feline epilepsy (Barnes Heller, 2018; Engel et al., 2017; Moore, 2014; Pakozdy et al., 2014, 2012) and a systematic review of ASDs in cats (Charalambous et al., 2018) are available. In summary, PB is the most recommended monotherapeutic ASD in both species and/or IMP in dogs, followed by KBr for dogs and LEV for cats as the second line, and then ZNS and LEV for dogs and ZNS, GBP, PGB, and diazepam (DZP) for cats as the third or fourth line.

However, the selection and availability of ASDs are different in each country and may be influenced by many external factors such as economic, geographical, historical and commercial backgrounds and also owners’ compliance (including cost, ease of administration, motivation, education for treatment of their own animals and the owner's lifestyle).

Therefore, in order to grasp the current status of ASD usage in dogs and cats with epilepsy in Japan, we conducted a questionnaire survey of Japanese veterinary hospitals. The authors designed and made the questionnaire items (summarized in Table 1; the complete version is provided in Supplementary Table 1), and the survey was conducted by a third‐party organization (Zpeer, Inc., Japan; https://zpeer.info/en/ ), which provides professional veterinary information on the Internet that veterinarians or veterinary hospitals can register for as members for free. Recruitment was conducted by sending a direct mail to 5,421 hospitals registered as a director of their veterinary hospital; the number of veterinary clinics for small animals in Japan in 2020 was 12,247 according to Japan's Ministry of Agriculture, Forestry and Fisheries. The survey period was 7 days (18–24 September 2020).

**TABLE 1:** Summary of the questionnaire

Questions
Which oral antiseizure drugs does your hospital currently hold? (Multiple answers allowed)
What are the top three oral antiseizure drugs prescribed for dogs or cats with idiopathic or structural epilepsy? (most prescribed, second‐most prescribed, third‐most prescribed)
What combination of two oral antiseizure drugs is prescribed for dogs with idiopathic epilepsy?
When prescribing phenobarbital, zonisamide and potassium bromide, do you measure blood concentration?
If so, how often? And if not, why? (Multiple answers allowed)
How many canine/feline patients were newly diagnosed with idiopathic or structural epilepsy per year? (The average for the last 3–5 years)
How many canine/feline patients with idiopathic or structural epilepsy have been treated continuously since the previous year? (The average for the last 3–5 years)

The complete version is displayed in Supplementary Table 1.

Finally, we received responses from 511 veterinary hospitals (response rate: 9.4%), and all respondents were the director and answered on behalf of each hospital. Approximately 60% of those hospitals had only one veterinarian (director). The results are summarized in Tables 2 and 3 and Figures 1, 2, 3, 4 (see Supplementary Table 2 for details of Figures 2 and 4). As a remarkable result of this survey, most of the hospitals held ZNS (97.7%) and ZNS was used as the most frequently prescribed ASD for dogs with idiopathic (83.4%) and structural epilepsy (75.9%) (Figure 2). Additionally, ZNS was the most frequently used ASD in combination therapy (Figure 3). In cats, although PB was used most frequently for idiopathic (47.8%) and structural (50.7%) epilepsy, ZNS was also used frequently (40.5% and 36.2%, respectively) (Figure 4).

**TABLE 2:** The characteristics of Japanese veterinary hospitals

	Average	Median
New canine patients diagnosed with idiopathic or structural epilepsy per year	4.1	3
Canine patients with idiopathic or structural epilepsy who have been treated continuously since the previous year	7.6	5
New feline patients diagnosed with idiopathic or structural epilepsy per year	1.3	1
Feline patients with idiopathic or structural epilepsy who have been treated continuously since the previous year	2.0	1

**TABLE 3:** The answers for when ASD blood concentrations were measured

Performed at the first time and at the time of dose change	45.79%
Performed when adverse effects appear	25.64%
Not done	22.11%
Reasons why: They did not feel the necessity for measuring blood concentration: 49.56% The owner's financial limitations: 25.66% Measuring blood concentration took time and effort: 12.39% Other: 12.39%	
Performed regularly	16.24%
Every 1 month: 2.40% Every 2 months: 3.61% Every 3 months: 20.48% Every 4 months: 4.82% Every 6 months: 56.63% Every 12 months: 12.05%	
Other	7.44%

**FIGURE 1:**
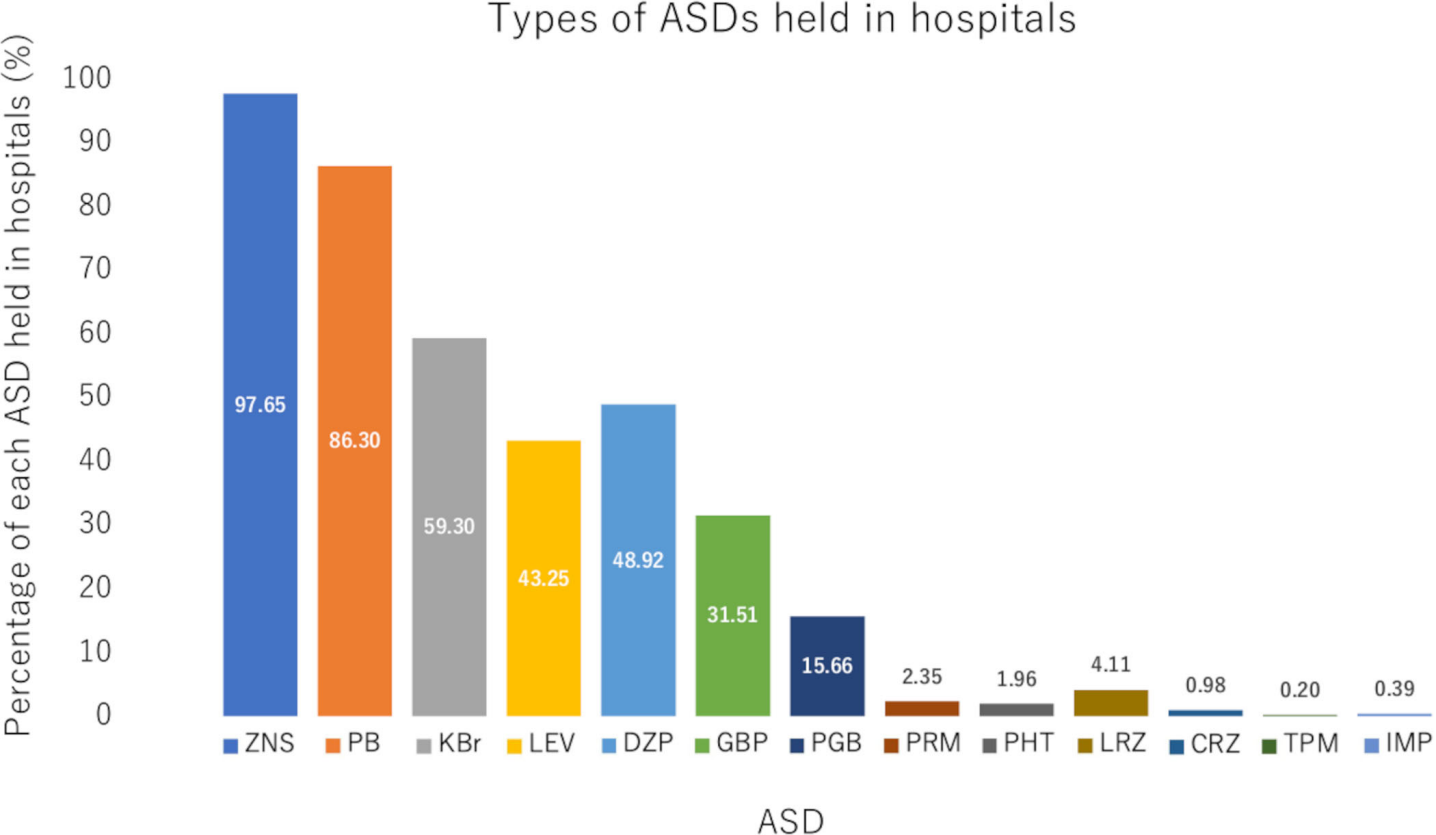
The types of ASD held in Japanese veterinary hospitals. DZP, diazepam; GBP, gabapentin; IMP, imepitoin; KBr, potassium bromide; LEV, levetiracetam; LRZ, lorazepam; PB, phenobarbital; PGB, pregabalin; PHT, phenytoin; PRM, primidone; TPM, topiramate; ZNS, zonisamide

**FIGURE 2:**
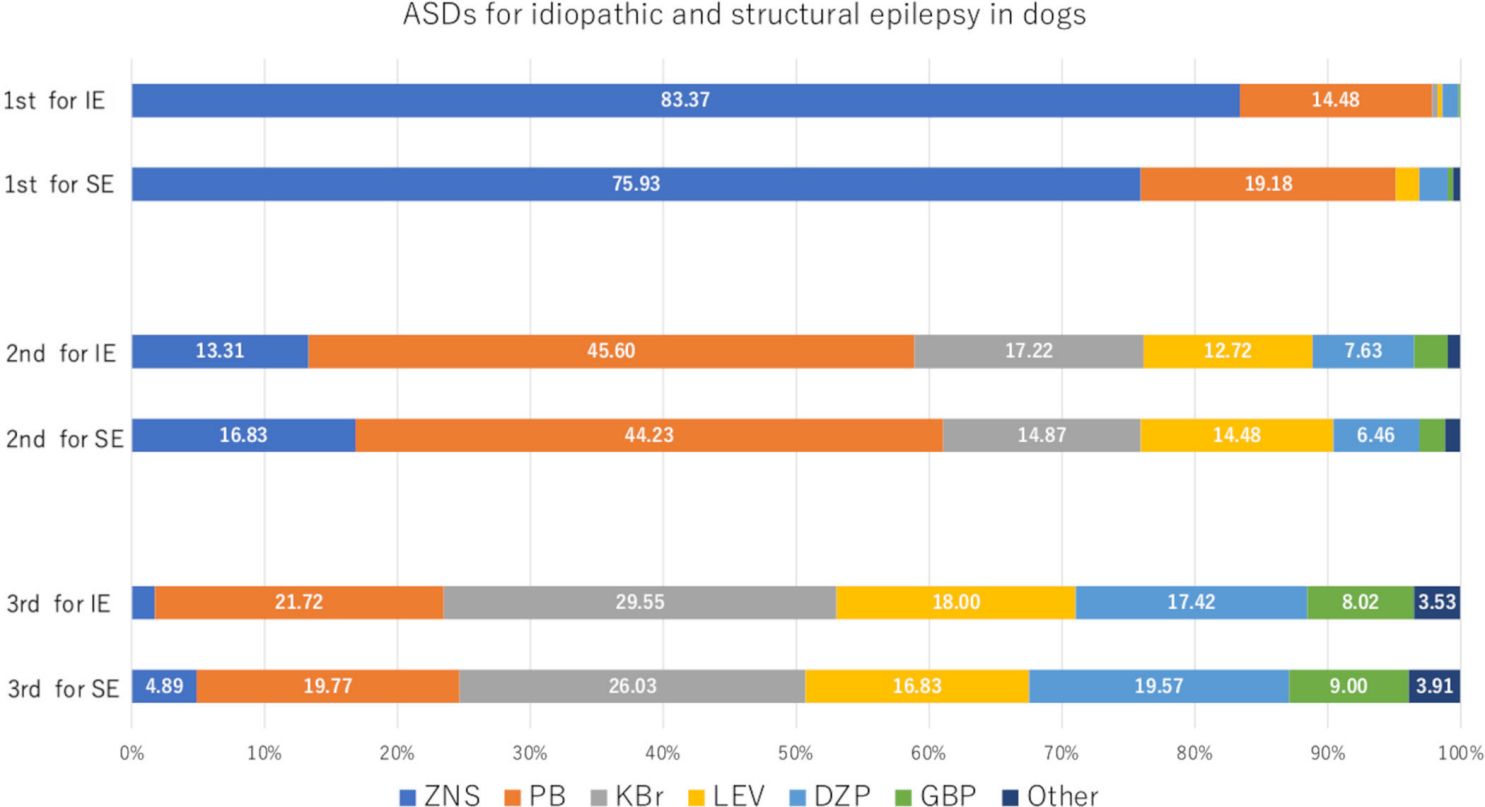
ASD usage for dogs with idiopathic and structural epilepsy in Japanese veterinary hospitals. Detailed data are available in Supplementary Table 2. IE, idiopathic epilepsy; SE, structural epilepsy; 1^st^, the most prescribed drug; 2^nd^, the second‐most prescribed drug; 3^rd^, the third‐most prescribed drug. Abbreviations for ASD are the same as in Figure 1

**FIGURE 3:**
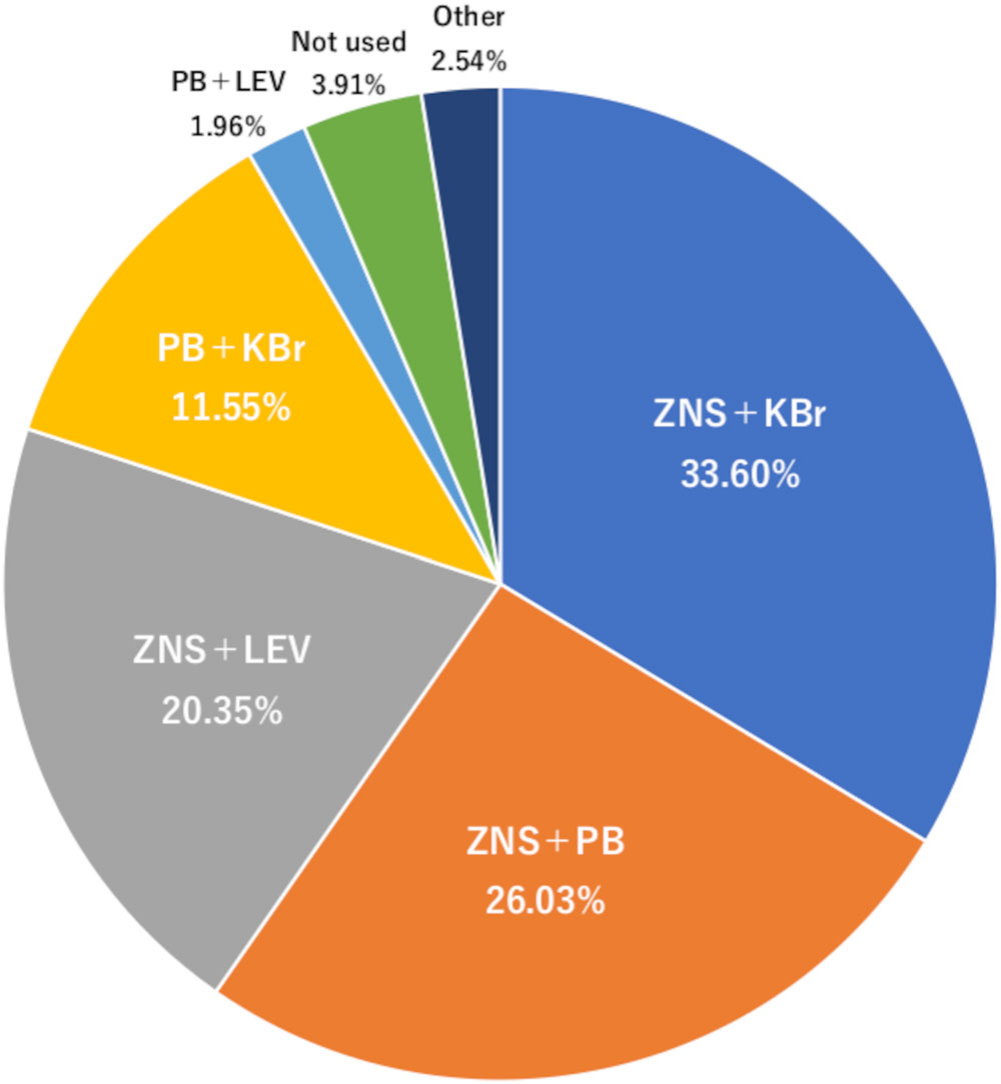
The combined usage of two ASDs for dogs with idiopathic epilepsy in Japanese veterinary hospitals. Abbreviations for ASD are the same as in Figure 1

**FIGURE 4:**
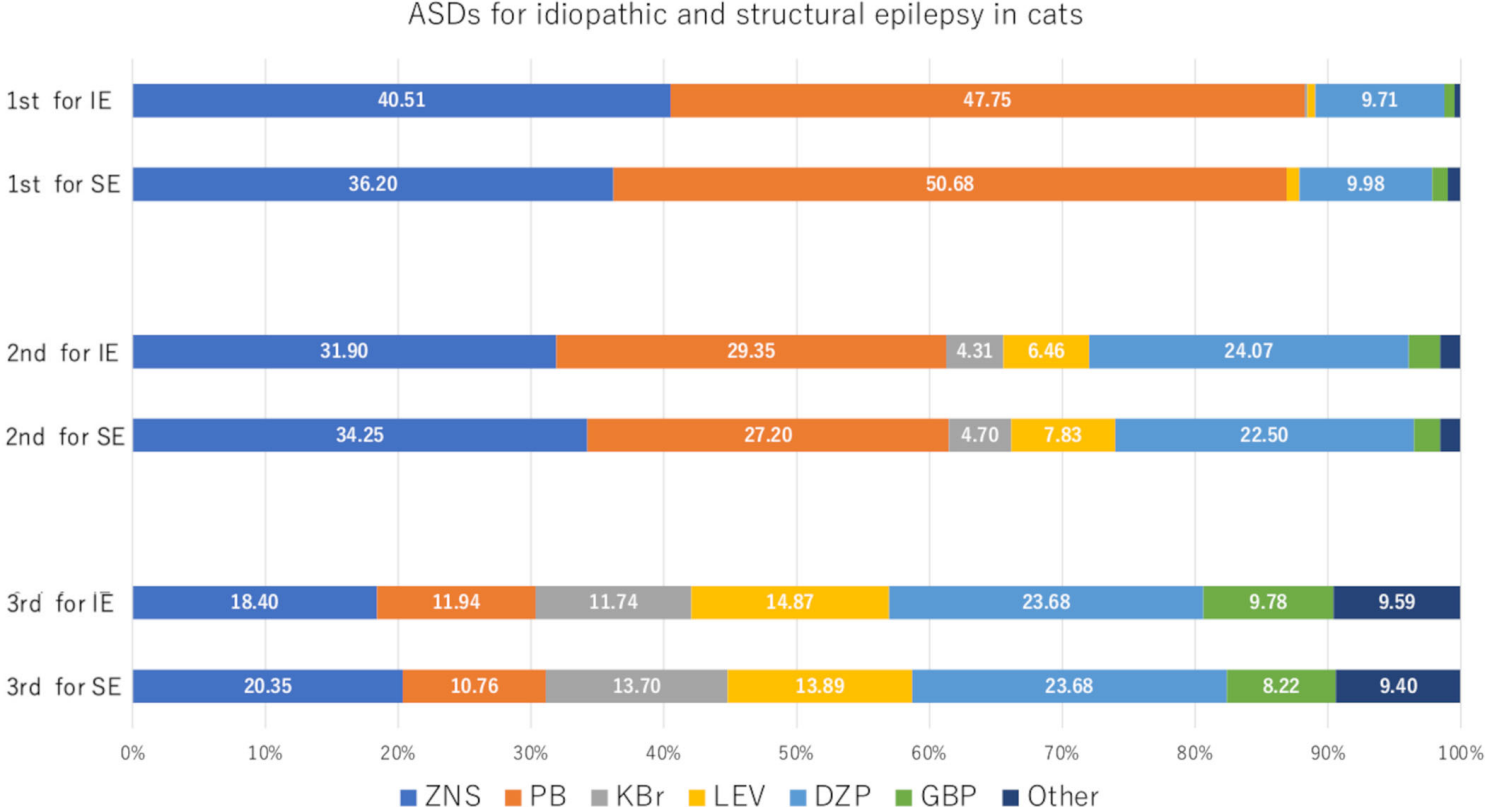
ASD usage for cats with idiopathic and structural epilepsy in Japanese veterinary hospitals. Detailed data are available in Supplementary Table 2. Abbreviations are the same as in Figure 2

Although most veterinary hospitals measured ASD blood levels at initiation, dose changes and the occurrence of adverse effects, another noteworthy result is that more hospitals (22.1%) did not measure ASD blood levels at all compared to those that did regularly (16.2%) (Table 3). The main reasons for not measuring ASD blood levels were that Japanese clinicians did not feel it necessary (49.6%) and owners’ financial limitations (25.7%).

Despite the fact that the consensus reports in dogs and a systematic review in cats did not recommend ZNS as a first‐line drug for either species, this survey revealed that ZNS was being used overwhelmingly more frequently in Japan than in Western countries. Potential reasons for this include that ZNS was originally developed in Japan in the 1980s and has a long history of use not only in human medicine but also in veterinary medicine, and Japanese veterinarians have rarely experienced serious adverse effects with ZNS in their long history of its usage. These experiences facilitated the licensing of ZNS as the first ASD for dogs in Japan (2015). At that time, ZNS was widely campaigned to Japanese veterinarians. Conversely, these factors may have led Japanese veterinarians to misunderstand the effectiveness and safety of ZNS (and PB) in cats with epilepsy. In cats, the adverse effects of ZNS are more likely to occur at lower blood concentrations than in dogs (Hasegawa et al., 2008; Matsuda et al., 1979), and PB is highly valued for both its safety and efficacy in cats, and rarely causes hepatic enzyme induction or biochemical abnormalities (Charalambous et al., 2018; Hermans et al., 2021). Additionally, IMP was also licenced in Japan for dogs in 2015; however, it has not been sold so far.

Furthermore, this survey revealed the frequency of DZP (10–24% in dogs; Figure 2) and KBr (0–14% in cats; Figure 4) use in Japanese veterinary hospitals. Chronic oral administration of DZP is not recommended for dogs because of its short half‐life and the development of tolerance (Podell, 2013). Similarly, the use of KBr in cats is not recommended because it reportedly causes a relatively high rate of bronchial asthma, which is sometimes fatal (Boothe et al., 2002). Although these drugs were not used frequently in this survey, some Japanese veterinarians may have misunderstand their appropriate use.

According to the concept of therapeutic drug monitoring (TDM), measurements of ASD blood (serum or plasma) concentrations should be performed under the following circumstances: (1) when a steady state has been reached after initiation, the dose is changed, and/or a loading dose is given; (2) when seizures are not controlled despite an adequate dose; (3) when ASD‐related toxicity (adverse effect) is suspected; and (4) when regularly checking for changes in drug activity every 6 (PB and ZNS) to 12 (KBr) months, as recommended in IVETF and ACVIM consensus statements (Bhatti et al., 2015; Podell et al., 2016). Although it is ideal that TDM is conducted for all ASDs, TDM in veterinary medicine has been recommended in ASDs that have a certain half‐life such as PB, KBr, and ZNS. In particular, when used in combination with PB, the pharmacokinetics of ZNS are changed (Hojo et al., 2002; Orito et al., 2008), so practitioners should pay attention to its levels. ASDs are often taken for the rest of a patient's life and can be an economic burden on the owner; however, if we do not follow TDM, different ASDs may be prescribed before the initial treatment has shown a sufficient effect or may cause more adverse effects than necessary. Therefore, clinicians should measure blood levels as much as possible for adequate and successful ASM. On the other hand, DZP and new generated ASDs such as LEV, GBP, PGB and IMP do not require TDM due to their unique profiles including short half‐life, safety and unestablished correlation between concentration and efficacy or toxicity (Bhatti et al., 2015; Podell et al., 2016).

The limitation of this study was that the determination of prescription frequency order was entrusted to each hospital; therefore, selection criteria were not certain. Furthermore, only the director responded to this questionnaire, thus in hospitals where many veterinarians worked, there may be variations among clinicians. We note that these results were based on a survey of prescribing frequency, not prescribing priority; that may not accurately reflect the meaning of the order of the first, second and subsequent lines of ASM. In addition, we did not investigate the detailed frequency of prescriptions, long‐term therapeutic efficacy and safety of each ASD. Therefore, further studies including these factors, such as a large‐scale examination of ZNS or IMP (or other newer ASDs) in dogs and cats in Japan or worldwide, may change the future recommendations for ASM.

To the best of our knowledge, this is the first report to investigate the actual situation of ASM for dogs and cats with epilepsy in Japan. In this survey, we revealed the peculiarities of ASD usage in Japan and also learned the importance of continuously educating veterinarians with current information about the efficacy and safety of each ASD and adequate ASM including TDM in each species. Veterinary neurologists, pharmacologists and academics should make the effort to perform research to build up evidence for appropriate ASM, while general practitioners should always be up to date with the latest and correct knowledge.

## CONFLICT OF INTEREST

The authors’ laboratory received a donation for academic research from Sumitomo Pharma Animal Health Co., Ltd. (former DS Pharma Animal Health Co., Ltd).

## ETHICS STATEMENT

No ethical approval was required as no animals were used.

## AUTHOR CONTRIBUTION

Satoshi Mizuno: Data curation, Visualization, Writing ‐ original draft; Daisuke Hasegawa: Conceptualization, Methodology, Project administration, Writing ‐ review & editing.

### PEER REVIEW

The peer review history for this article is available at https://publons.com/publon/10.1002/vms3.810.

## Supporting information

Table S1

Table S2

## Data Availability

The data that support the findings of this study are available from the corresponding author (DH) upon reasonable request.
