# Supplementary material for: Questionnaire survey on the usage of antiseizure drugs for dogs and cats in Japanese veterinary hospitals (2020)
Source: Vet Med Sci. 2022 Apr 20;8(4):1466–71. doi: 10.1002/vms3.810 (PMC9297804; doi:10.1002/vms3.810)
Supplement: Supplementary file 1 — Table S1 [file VMS3-8-1466-s001.docx]

**Supplementary Table 1.** Questionnaire (complete version).

Questionnaire

- Which oral antiseizure drugs does your hospital hold currently? (Multiple answers allowed)
- Phenobarbital
- Zonisamide
- Potassium bromide
- Levetiracetam
- Diazepam
- Gabapentin
- Pregabalin
- Primidone
- Phenytoin
- Lorazepam
- Clonazepam
- Clobazam
- Other (enter the drugs: )
- What are the top three oral antiseizure drugs prescribed for DOGs with IDIOPATHIC epilepsy?
- Most prescribed:
- Second-most prescribed:
- Third-most prescribed:
- What are the top three oral antiseizure drugs prescribed for DOGs with STRUCTURAL epilepsy?
- Most prescribed:
- Second-most prescribed:
- Third-most prescribed:
- What are the top three oral antiseizure drugs prescribed for CATs with IDIOPATHIC epilepsy?
- Most-prescribed:
- Second-most prescribed:
- Third-most prescribed:
- What are the top three oral antiseizure drugs prescribed for CATs with STRUCTURAL epilepsy?
- Most-prescribed:
- Second-most prescribed:
- Third-most prescribed:
- What is the combination of the two oral antiseizure drugs prescribed for DOGs with IDIOPATHIC epilepsy?
- Phenobarbital + Potassium bromide
- Zonisamide + Potassium bromide
- Phenobarbital + Zonisamide
- Phenobarbital +Levetiracetam
- Zonisamide + Levetiracetam
- Other combination ( + )
- When prescribed Phenobarbital, Zonisamide and Potassium bromide, do you measure blood concentration? If so, how often? And if not, why? (Multiple answers allowed)
- Not done

The reason why ( )

- Performed at the first time and at the time of dose change
- Performed when adverse effects appear
- Performed regularly

Every ( ) month(s)

- Other ( )
- How many CANINE patients are newly diagnosed with idiopathic or structural epilepsy per year? (average for the last 3-5 years)
- ( ) canine patient(s)
- How many CANINE patients with idiopathic or structural epilepsy have been treated continuously since the previous year? (average for the last 3-5 years)
- ( ) canine patient(s)
- How many FELINE patients are newly diagnosed with idiopathic or structural epilepsy per year? (average for the last 3-5 years)
- ( ) feline patient(s)
- How many FELINE patients with idiopathic or structural epilepsy have been treated continuously since the previous year? (average for the last 3-5 years)
- ( ) feline patient(s)

About you

- Your generation
  - 26–29
  - 30–39
  - 40–49
  - 50–59
  - Over 60
- How many veterinarians work in your hospital?
  - ( ) vet(s)
