# Supplementary material for: Questionnaire survey on the usage of antiseizure drugs for dogs and cats in Japanese veterinary hospitals (2020)
Source: Vet Med Sci. 2022 Apr 20;8(4):1466–71. doi: 10.1002/vms3.810 (PMC9297804; doi:10.1002/vms3.810)
Supplement: Supplementary file 2 — Table S2 [file VMS3-8-1466-s002.docx]

**Supplementary Table 2.**

The breakdown of the top three oral antiseizure drugs prescribed for dogs and cats with idiopathic or structural epilepsy in the survey (complete version).

**ASDs for idiopathic and structural epilepsy in dogs**

|  | 1^st^ for IE | 1^st^ for SE | 2^nd^ for IE | 2^nd^ for SE | 3^rd^ for IE | 3^rd^ for SE |
| --- | --- | --- | --- | --- | --- | --- |
| ZNS | 83.37 | 75.93 | 13.31 | 16.83 | 1.76 | 4.89 |
| PB | 14.48 | 19.18 | 45.60 | 44.23 | 21.72 | 19.77 |
| KBr | 0.39 | 0 | 17.22 | 14.87 | 29.55 | 26.03 |
| LEV | 0.39 | 1.76 | 12.72 | 14.48 | 18.00 | 16.83 |
| DZP | 1.17 | 2.15 | 7.63 | 6.46 | 17.42 | 19.57 |
| GBP | 0.20 | 0.39 | 2.54 | 1.96 | 8.02 | 9.00 |
| Other | 0 | 0.59 | 0.98 | 1.17 | 3.53 | 3.91 |

**ASDs for idiopathic and structural epilepsy in cats**

|  | 1^st^ for IE | 1^st^ for SE | 2^nd^ for IE | 2^nd^ for SE | 3^rd^ for IE | 3^rd^ for SE |
| --- | --- | --- | --- | --- | --- | --- |
| ZNS | 40.51 | 36.20 | 31.90 | 34.25 | 18.40 | 20.35 |
| PB | 47.75 | 50.68 | 29.35 | 27.20 | 11.94 | 10.76 |
| KBr | 0.20 | 0 | 4.31 | 4.70 | 11.74 | 13.70 |
| LEV | 0.59 | 0.98 | 6.46 | 7.83 | 14.87 | 13.89 |
| DZP | 9.71 | 9.98 | 24.07 | 22.50 | 23.68 | 23.68 |
| GBP | 0.78 | 1.17 | 2.35 | 1.96 | 9.78 | 8.22 |
| Other | 0.46 | 0.99 | 1.56 | 1.56 | 9.59 | 9.40 |

Abbreviation: for IE, for idiopathic epilepsy; for SE, for structural epilepsy; 1^st^, the most prescribed drug; 2^nd^, the second-most prescribed drug; 3^rd^, the third-most prescribed drug; DZP, diazepam; GBP, gabapentin; KBr, potassium bromide; LEV, levetiracetam; PB, phenobarbital; ZNS, zonisamide.
